# Draft genome sequence of *Escherichia coli* LA3 isolated from influent wastewater discharge of a tertiary-level hospital in Laguna, Philippines

**DOI:** 10.1128/mra.00921-25

**Published:** 2026-03-19

**Authors:** Shaughn Dominic M. Padon, Erica Mai T. Cabuyao, Nathaniel John Q. Cruz, Kiara Nicole D. Rodriguez, Leslie Michelle M. Dalmacio, Liza Bautista-Patacsil, Arnel B. Beltran, Aileen H. Orbecido, Joel C. Cornista

**Affiliations:** 1 Department of Biology, College of Arts and Sciences, University of the Philippines Manila 54725, Manila, Philippines; 2 Department of Biochemistry and Molecular Biology, College of Medicine, University of the Philippines Manila 54725, Manila, Philippines; 3 Department of Engineering Science, College of Engineering and Agro-Industrial Technology, University of the Philippines Los Baños 54729, Laguna, Philippines; 4 Department of Chemical Engineering, Gokongwei College of Engineering, De La Salle University 63148, Manila, Philippines; University of Maryland Baltimore, Baltimore, Maryland, USA

**Keywords:** extraintestinal pathogenic *Escherichia coli*, wastewater treatment, urinary tract infections, multidrug resistance, *qacEΔ1*

## Abstract

Hospital wastewater-associated strains of *Escherichia coli* are increasingly recognized as reservoirs of virulence and antimicrobial resistance determinants with potential implications for nosocomial infections. In this study, we report the draft genome sequence of *E. coli* strain LA3, isolated from untreated influent wastewater discharged by a tertiary-level hospital in Laguna, Philippines.

## ANNOUNCEMENT

*Escherichia coli*, a ubiquitous coliform, proliferates in hospital wastewater alongside other nosocomial pathogens (1, 2). Extraintestinal pathogenic *E. coli* lineages primarily drive urinary tract infections and disseminate antimicrobial resistance genes (ARGs) (3–5). Consequently, genomic characterization of multidrug-resistant (MDR) *E. coli* LA3 offers insights into the environmental persistence of drug-resistant bacteria within hospital wastewater systems.

LA3 was isolated from influent wastewater (14.1769°N, 121.2730°E) of a tertiary-level hospital in Laguna, Philippines. One liter samples, retrieved in triplicates from a depth of 15–20 ft, were transported (4°C) to Microbiology Laboratory, Department of Biology, University of the Philippines (UP) Manila. The pooled sample was serially diluted in 0.85% (wt/vol) saline solution, spread-plated onto nutrient agar (HiMedia, India), and incubated (30°C, 24 h). Ten randomly selected colonies were phenotypically screened against 14 antibiotics (Table 1) (6). MDR isolate LA3 was selected for whole-genome sequencing, exhibiting high multiple antibiotic resistance index (0.71) (7).

**TABLE 1:** Scaffold-level assembly characteristics, phenotypic resistance, and plasmid profile of *Escherichia coli* LA3

General features	
% Genome completeness	98.45
% Genome contamination	2.47
Paired-end reads	2 × 877,917
Genome size (Mbp)	5.3
Number of contigs	468
Number of scaffolds	463
Number of plasmids	9
% G + C content	50.5
Genome coverage	41.1×
Scaffold N_50_ (kbp)	111.9
Scaffold L_50_	16
Coding sequences (CDS)	3,617
CDS ratio	0.99
Pseudogenes	38
RNAs	141
rRNA	46
tRNA	81
ncRNA	4
Pathogenicity likelihood (%)	97.42 ± 1.07

Group	Antibiotic	Disk content (mg)	Zone diameter interpretative standards (mm)^*a*^		
			Susceptible	Immediate	Resistant
Common clinical antibiotics	Clarithromycin	15	≥13	11–12	≤10
	Azithromycin	30	≥13	12.1–12.9	≤12
	Metronidazole	50	≥32	9–31	≤8
	Ciprofloxacin	30	≥21	16–20	≤15
	Levofloxacin	5	≥17	14–16	≤13
Cephalosporin antibiotics	Cefotaxime	30	≥23	15–22	≤14
	Cefuroxime	30	≥18	15–17	≤14
	Ceftazidime	10	≥18	15–17	≤14
	Cefazolin	30	≥15	14.1–14.9	≤14
ESBLs and alternatives	Ampicillin	25	≥17	14–16	≤13
	Piperacillin	75	≥21	18–20	≤17
	Clindamycin	10	≥21	15–20	≤14
	Vancomycin	30	≥17	15–16	≤14
	Meropenem	20	≥16	14–15	≤13

Plasmid ID	Replicon types	Size (bp)	G + C content (%)	Mobility^*b*^	Antimicrobial resistance genes
AA860	IncC	187,740	52.72	C	qacEΔ1 Sul1 arr-2 cmlA5 bla_OXA-10_ bla_TEM-1_ bla_CTXM-55_ QnrS1 APH(6)-Id APH(3″)-lb APH(3″)-la sul2
AB130	–^*c*^	68,477	46.65	NM	–
AA378	IncY	95,090	47.89	NM	–
AA327	IncFIA IncN	86,495	50.87	C	FosA3
AB717	IncX1	9,090	41.00	NM	–
AB229	–	6,379	55.76	NM	–
AA316	IncFIA IncFIC	37,898	50.72	C	–
AF232	–	4,155	55.38	NM	–
AA537	ColRNAI	4,145	52.79	M	–

^*a*^ Antibiogram profile of LA3 isolate highlighted in gray, with interpretative standards based on CLSI M100 disk diffusion standards (6). ESBL, extended-spectrum beta-lactamase.

^*b*^ Mobility classifications: C, conjugative; M, mobilizable; NM, non-mobilizable. Replicon types were identified via the MOB-Recon 3.1.9+galaxy0 (8).

^*c*^ –, no data available.

After several rounds of subculturing, bacterial cells from an isolated colony were grown overnight (30°C) in 5 mL nutrient broth (HiMedia). Genomic DNA was extracted using HiPurA Bacterial Genomic DNA Purification Kit (HiMedia), following the manufacturer’s protocol. Concentration and purity (*A*_260_/*A*_280_) of gDNA were determined using LVis plate on FLUOstar Omega (BMG LABTECH, Germany) multimode microplate reader. Sequencing was done at the DNA Sequencing Core Facility, Philippine Genome Center, UP Diliman (Quezon City, Philippines). TruSeq DNA Nano Kit (Illumina, USA) was used for library construction using sample gDNA (19.3 ng/µL), sheared (focused ultrasonication), and bead-selected (508 bp library size). Samples were barcode-indexed and pooled for multiplexing, preceding Illumina MiSeq sequencing (2 × 250 bp paired-end chemistry).

Default parameters were used for all software unless otherwise specified. Trimmomatic 0.39 (9) was used to control raw read quality (ILLUMINACLIP:TruSeq3-PE.fa:2:30:10 LEADING:3 TRAILING:3 SLIDINGWINDOW:4:15 MINLEN:36) and SPAdes 4.20 (10) for *de novo* assembly (--careful --k21,33,55,77). Scaffold quality was evaluated using QUAST 5.2.0 (11) and gVolante 2 (12) (BUSCO v5, genome mode). The genome was annotated using NCBI Prokaryotic Genome Annotation Pipeline 6.1 (13). The isolate was identified using KmerFinder 3.2 (14–16), then confirmed using pairwise average nucleotide identity (ANI) analysis using BLAST-based ANIb 4.0.1 (95% similarity threshold) in JSpeciesWS server (17, 18). Multilocus sequence typing was performed using MLST 2.0 (19), serotyping with SerotypeFinder 2.0 (20), and phylotyping with ClermonTyper 20.03 (21, 22). Putative plasmids were reconstructed and typed using MOB-Recon 3.1.9+galaxy0 (8). ARGs were identified using CARD RGI 6.0.3 (23), excluding loose and nudged hits. Human-pathogenic potential was predicted using PathogenFinder2 0.5.0 (24).

LA3, identified as *E. coli* (83.72% query coverage vs. 2014-01-7375^T^) and confirmed by pairwise ANI (99.0% similarity with KOEGE 131 [358a], NCBI:txid1281172), harbors nine identified plasmids (Table 1). The isolate is of sequence type (ST) 453, under *E. coli* phylogroup B1, associated with environmental lineages (3). Isolate serotype O23:H16 is implicated in clinical reservoirs (25). Assembly statistics are summarized in Table 1.

## Data Availability

The draft genome assembly and raw reads were deposited in NCBI GenBank under accession numbers GCA_052046135.1 and SRR34895959, respectively. Both of these were deposited under BioProject PRJNA1302472.
